# The Treatment of Acute Inflammatory Disease of the Heart

**Published:** 1898-04-09

**Authors:** 


### THE TREATMENT OF ACUTE INFLAMMATORY DISEASE OF THE HEART.

In the Lumleian Lectures Sir R. Douglas Powell lays down a general broad rule in regard to the treatment of acute inflammatory diseases of the heart, which, while true enough to be of great use in daily practice, must be taken as it was given, namely, as applying to the general run, and not to those exceptional cases, the early recognition of which is the highest proof of the acumen of the physician. Still, broad, safe rules have their uses. What Sir Douglas Powell says is to this effect: The general line of treatment of acute inflammatory diseases of the heart is the same in the vast majority of cases. We might, after recognising the presence of heart disease, so far as treatment is concerned, keep our stethoscopes in our pockets, for we have chiefly to consider the fundamental complaint of which the cardiac affection is in most cases a part, and this disease is, in the majority of cases, rheumatism. From the heart point of view the right treatment of rheumatism is absolute rest in bed in woollen wrappings, a free relief of the bowels, and the administration of salicylate of soda in efficient doses in combination with such alkaline remedies as the condition of the urine may suggest. Few cases at the present day have to endure more than five days' suffering under the salicylate treatment, and a line of treatment so successful in regard to the general disease can hardly fail to prove in some degree preventive of cardiac lesions. It can scarcely be doubted that the current view that salicylates do not lessen the liability to cardiac lesions arises from the fact that cases early relieved of pain and fever by salicylates are allowed to get up too soon, and thus fall into the category of those not treated by rest. The average time spent in hospital for acute rheumatism (thirty days) is nearly the same under all treatments, and during the greater part of this time the patient must remain in bed under strict treatment by warmth and rest and appropriate drugs. If then, on the relief of pain and reduction of temperature, the patient be regarded as convalescent and allowed out of bed, he becomes doubly liable to cardiac manifestations. As to the local treatment of cardiac disease in rheumatism, it cannot be said that any one plan is to be recommended as superior in all cases. It is for diagnosis and prognosis that the stethoscope is required. Especially is this the case in any febrile complaint in an infant or child until its nature is fully declared, for cardiac rheumatism in the young is frequently associated with little or no joint manifestations.

